# The “Real-World” Effect of Anti-hyperglycemic Drugs on the Development of Chronic Kidney Disease in a Retrospective Cohort of Patients With Incident Diabetes: A Research Letter

**DOI:** 10.1177/20543581251365364

**Published:** 2025-08-22

**Authors:** Anita Dahiya, Ming Ye, Jennifer E. Vena, Grace Shen Tu, Jeffrey A. Johnson, Dean T. Eurich

**Affiliations:** 1 School of Public Health, University of Alberta, Edmonton, Canada; 2 Alberta’s Tomorrow Project, Cancer Care Alberta, Alberta Health Services, Calgary, Canada

**Keywords:** chronic kidney disease, anti-hyperglycemic drugs, incident diabetes, retrospective

## Abstract

Recent clinical trials suggest benefit of anti-hyperglycemic drugs on kidney outcomes. However, there is a paucity of information available on the real-world impact.We aimed to study the real-world impact of anti-hyperglycemic drugs (metformin, sodium-glucose cotransporter-2 (SGLT-2) inhibitors, dipeptidyl peptidase-4 (DPP-4) inhibitors, and glucagon-like peptide-1receptor (GLP-1R) agonists) using a cohort of patients with incident diabetes derived from the Alberta Tomorrow Project (ATP) database. A retrospective cohort was created from the ATP database using administrative data from October 1, 2000, to March 31, 2021. We examined the effect of anti-hyperglycemic medications including metformin (as a control), SGLT-2 inhibitors, DPP-4 inhibitors, and GLP-1R agonists on a composite kidney outcome including chronic kidney disease, kidney failure, dialysis, kidney transplant, and kidney-related death using a Cox-regression analysis.

The study included 3001 patients with an incident diagnosis of diabetes. The average follow-up was 6.7 ± 4.6 years after diagnosis, and 628 (20.9%) patients reached the composite outcome with a mean of 5.6 ± 4.2 years to the first event. A total of 1749 (58.8%) patients were on metformin, 360 (12.0%) on SGLT-2 inhibitors, 313 (10.4%) on DPP-4 inhibitors, and 188 (6.3%) on GLP-1R agonists. Only the patients prescribed SGLT-2 inhibitors had a significant reduction in the composite outcome (hazard ratio (HR) 0.23, 95% CI 0.09-0.62, *P*-value = .003), and a dose-related effect was observed. Our study has shown that SGLT-2 inhibitors result in significant reduction of composite kidney outcomes, including chronic kidney disease, suggesting a renally protective effect over long term.

## Abrégé

De récents essais cliniques suggèrent un bienfait des médicaments antihyperglycémiques sur les résultats de santé rénale. Il existe cependant peu d’informations disponibles sur leur impact réel. Notre objectif était d’étudier l’impact réel des médicaments antihyperglycémiques (metformine, inhibiteurs du SGLT-2, inhibiteurs de la dipeptidyl peptidase-4 [DPP-4] et agonistes du récepteur GLP-1 [glucagon-like peptide-1R]) dans une cohorte de patients diabétiques incidents dérivée de la base de données Alberta Tomorrow Project (ATP). Une cohorte rétrospective a été créée à partir des données administratives de l’ATP du 1^er^ octobre 2000 au 31 mars 2021. Nous avons examiné l’effet de médicaments antihyperglycémiques, soit la metformine (témoin), les inhibiteurs du SGLT-2, les inhibiteurs de la DPP-4 et les agonistes du GLP-1R, sur une issue composite de santé rénale telle que l’insuffisance rénale chronique, l’insuffisance rénale terminale, la dialyze, la transplantation rénale et le décès lié à une néphropathie, en utilisant une analyze de régression de Cox.

L’étude a inclus 3 001 patients avec un diagnostic incident de diabète. Le suivi moyen était de 6,7±4,6 ans après le diagnostic. Un total de 628 patients (20,9 %) a évolué vers une issue rénale composite 5,6±4,2 ans en moyenne avant le premier événement. Dans la cohorte, 1749 patients (58 %) prenaient de la metformine, 360 (12,0 %) des inhibiteurs du SGLT-2, 313 (10,4 %) des inhibiteurs de la DPP-4 et 188 (6,3 %) des agonistes du GLP-1R. Seuls les patients traités par des inhibiteurs du SGLT-2 ont présenté une réduction significative du critère de jugement composite (RR: 0,23; IC 95 %: 0,09 à 0,62; p = 0,003) avec un effet dépendant de la dose. Notre étude montre que les inhibiteurs du SGLT-2 entraînent une réduction significative des issues cliniques rénales composites, y compris l’insuffisance rénale chronique, ce qui suggère un effet protecteur à long terme.

## Introduction

Diabetic kidney disease is the leading cause of chronic kidney disease worldwide, accounting for 50% of cases. Although there has been a decrease in incidence, nearly half of new dialysis starts in Canada are in patients with diabetes.^1,2^ Recent clinical trials suggest potential benefits of anti-hyperglycemic drugs on kidney outcomes.^3,4^ Most notably, meta-analyses of these trials have shown sodium-glucose cotransporter-2 (SGLT-2) inhibitors were associated with improved albuminuria, reduced risk of kidney failure, and kidney-related death. However, varied results exist with several clinical trials showing no effect of anti-hyperglycemic drugs, including SGLT-2 inhibitors.^5^ Furthermore, there are limited pharmaco-epidemiology studies showing the real-world, long-term effect of anti-hyperglycemic drugs on kidney outcomes in a Canadian population.^6-8^

Thus, we aimed to study the real-world impact of anti-hyperglycemic drugs (metformin, SGLT-2 inhibitors, dipeptidyl peptidase-4 (DPP-4) inhibitors, and glucagon-like peptide-1receptor (GLP-1R) agonists) using a cohort of adult Albertan patients with incident diabetes derived from the Alberta Tomorrow Project (ATP) database from October 1, 2000, to March 31, 2021.

## Methods

Please see the Supplementary Methods for details of the study population. In summary, this retrospective cohort using data from the ATP cohort, linked with Alberta Health (AH; the provincial health ministry) administrative data from October 1, 2000, to March 31, 2021. We included adult patients with incident diabetes in the ATP cohort randomly recruited from Alberta’s adult general population in 2000 to 2008. We examined the effect of anti-hyperglycemic medications including metformin (as a control), SGLT-2 inhibitors, DPP-4 inhibitors, and GLP-1R agonists on a composite kidney outcome including chronic kidney disease, kidney failure, dialysis (hemodialysis and peritoneal dialysis), kidney transplant, and kidney-related death. Patients prescribed anti-hyperglycemic drugs 6 months prior to a diabetes diagnosis and/or 6 months prior to the outcome of interest were excluded to minimize the potential impact from use of drugs unrelated to diabetes and/or protopathic bias. Common doses of dapagliflozin (either 5 or 10 mg) were used as a rough estimate of the dose response effect for SGLT-2 inhibitors. Seventy-three of the 360 patients on SGLT-2 inhibitors were on dapagliflozin. Hazard ratios (HR) and its 95% confidence intervals were calculated after adjusting for time-related variation in use of oral anti-diabetic drugs, age at diabetes diagnosis, sex (male/female), ethnicity (European ancestry vs other), living in rural vs urban areas, education attainment (secondary or less, some post-secondary, post-secondary), body mass index (BMI) categories (<24.9 kg/m^2^, 25.0-29.9 kg/m^2^, >30.0 kg/m^2^), ever smoker (yes/no), number drinks of alcohol per day, physically active (yes/no, based on accumulating at least 210 minutes of moderate- to vigorous-intensity recreational physical activities per week in the past 12 months), tertiles of Healthy Eating Index Canada score for diet quality assessment, the number of Elixhauser comorbidities (0, 1-2, 2+ comorbidities) at diagnosis, time-varying measures of use of insulin and drugs for cardiovascular diseases (lipid-lowering agents, diuretics, beta blocker, calcium channel blocker, agents on renin-angiotensin system, and mineralocorticoid receptor antagonists) and hypertension.^9^ High-dimensional propensity scores (HDPS) for oral anti-diabetic drugs; HDPS was calculated based on data using 43 different dimensions.^10^ An additional analysis was conducted on the post-2014 cohort (n = 1435) to account for differences that may have occurred after the introduction of SGLT-2 inhibitors in 2014.

## Results

The study cohort included 3001 patients with an incident diagnosis of diabetes (Supplementary Table 1). The average follow-up was 6.7 ± 4.6 years after diagnosis, and 628 (20.9%) patients reached the composite outcome with a mean of 5.6 ± 4.2 years to the first event. Of the total population, 51.5% were female, and the average age was 61.3 ± 9.5 years at diagnosis. Among the study cohort, a total of 1749 (58.8%) patients were on metformin, 360 (12.0%) on SGLT-2 inhibitors, 313 (10.4%) on DPP-4 inhibitors, and 188 (6.3%) on GLP-1Ragonists. Patients that used anti-hyperglycemic medications were more likely to have no or 1 comorbidity. The top three comorbidities included hypertension (n = 2094, 69.8%), chronic pulmonary disease (n = 700, 23.3%), and depression (n = 687, 22.9%).

Unadjusted analysis of the composite outcome showed a significant reduction in cases irrespective of the anti-hyperglycemic drug prescribed (*P*-value < .05). HRs for age and ethnicity showed no significant difference in the composite outcomes (HR 1.12, 95% CI 0.83-1.51, *P* = .47 for patients aged 45-64 years old, HR 1.38, 95% CI 0.97-1.97, *P* = .07 for patients ≥65 years old, and HR 1.17, 95% CI 0.92-1.15, *P* = .21 for patients of European ancestry). However, once the data was adjusted for patient demographics, education status, BMI, lifestyle behaviors (alcohol use, physical activity, diet, and smoking), and other key medications (anti-hypertensive medications and drugs for cardiovascular diseases), only the patients prescribed SGLT-2 inhibitors had a significant reduction in the composite outcome (HR 0.23, 95% CI 0.09-0.62, *P*-value = .003; Table 1). This significance was retained after adjusting for HDPS (HR 0.15, 95% CI 0.05-0.40, *P* < .001). Furthermore, a dose-related effect was observed with higher doses of SGLT-2 inhibitors associated with lower rates of the composite outcomes (17.32 per 1000 person-years in patients prescribed 0-5 mg day vs 4.48 per 1000 person-years in patients prescribed 5 to 10 mg per day vs 1.89 per 1000 person-years in patients prescribed more than 10 mg per day). Additional analysis on the post-2014 cohort demonstrated similar results (HR 0.18, 95% CI 0.06-0.55, *P* < .05).

**Table 1.** Cox-Regression Analysis of Kidney-Related Outcomes After Prescription of Anti-Hyperglycemic Medications.

	Traditional model		HDPS	
	HR (95% CI)^a^	*P*-value	HR (95% CI)^b^	*P*-value
Met use yes (vs no)	1.17 (0.96-1.43)	0.12	1.15 (0.93-1.41)	.20
SLGT use yes (vs no)	0.23 (0.09-0.62)	0.003	0.15 (0.05-0.40)	<.001
DPP use yes (vs no)	0.88 (0.57-1.34)	0.54	0.72 (0.46-1.12)	.15
GLP use yes (vs no)	0.99 (0.43-2.27)	0.98	0.46 (0.19-1.13)	.09

a HR and its 95% CI was calculated after adjusting for time-related variation in use of oral anti-diabetic drugs, age at diabetes diagnosis, sex (male/female), ethnicity (European ancestry vs other), living in rural vs urban areas, education attainment (secondary or less, some post-secondary, post-secondary), BMI categories (<24.9 kg/m^2^, 25.0-29.9 kg/m^2^, 30.0 kg/m^2^), ever smoker (yes/no), number drinks of alcohol per day, physically active (yes/no, based on accumulating at least 210 minutes of moderate- to vigorous-intensity recreational physical activities per week in the past 12 months), tertiles of Healthy Eating Index Canada score for diet quality assessment, the number of Elixhauser comorbidities (0, 1-2, 2+ comorbidities) at diagnosis, time-varying measures of use of insulin and drugs for cardiovascular diseases (lipid-lowering agents, diuretics, beta blocker, calcium channel blocker, agents on renin-angiotensin system, and mineralocorticoid receptor antagonists), and hypertension.

b HR and its 95% CI was calculated after adjusting high-dimensional propensity scores (HDPS) for oral anti-diabetic drugs; HDPS was calculated based on data dimensions listed in the Supplementary Material.

## Discussion

Our study examining the real-world long-term effects of oral anti-hyperglycemic drugs on the development of chronic kidney disease in patients with diabetes has shown that SGLT-2 inhibitors result in a significant reduction of composite kidney outcomes, including chronic kidney disease, suggesting a renally protective effect over long term. This agrees with the short to mid-term data noted in multiple clinical trials. The same effect was not observed with other anti-diabetic medications. It is possible that the short-term beneficial effects of these anti-diabetic drugs observed in previous trials are not sustained over time; however, the mechanism for the same remains unclear. This study is strengthened by the adjustment for lifestyle factors such as smoking, alcohol use, physical activity, and diet; something which is not routinely captured in studies examining the effects of anti-diabetic medications. However, this study is limited by the low percentage of cases in patients using anti-hyperglycemic drugs that were relatively newly approved (2014 onwards) in Canada, such as SGLT-2 inhibitors, DDP-4 inhibitors, and GLP-1R agonists, which further limits the number of cases that can be assessed. Analysis of the post-2014 cohort demonstrated similar results, suggesting minimal impact from protopathic bias. Finally, we did not have access to patient drug coverage, and so it is possible there is a selection bias toward patients that can afford these anti-diabetic medications; however, we attempted to mitigate this by adjusting for various other sociodemographic factors. Further studies with larger cohorts are required to confirm the real-world long-term renal protective effect of SGLT-2 inhibitors over long term and why mechanistically the same effect is not observed in other anti-diabetic medications that have demonstrated short-term renal protective effects.

## Conclusion

Our study, although limited by its small sample size, has demonstrated that SGLT-2 inhibitors uniquely have a renally protective effect over long term in a retrospective cohort of patients with incident diabetes. Further studies with large cohorts are required to confirm the long-term effects of SGLT-2 inhibitors and why a similar long-term effect is not seen with DPP-4 inhibitors and GLP-1R agonists.

## Supplemental Material

sj-docx-1-cjk-10.1177_20543581251365364 – Supplemental material for The “Real-World” Effect of Anti-hyperglycemic Drugs on the Development of Chronic Kidney Disease in a Retrospective Cohort of Patients With Incident Diabetes: A Research Letter Supplemental material, sj-docx-1-cjk-10.1177_20543581251365364 for The “Real-World” Effect of Anti-hyperglycemic Drugs on the Development of Chronic Kidney Disease in a Retrospective Cohort of Patients With Incident Diabetes: A Research Letter by Anita Dahiya, Ming Ye, Jennifer E. Vena, Grace Shen Tu, Jeffrey A. Johnson and Dean T. Eurich in Canadian Journal of Kidney Health and Disease
