# Supplementary material for: The “Real-World” Effect of Anti-hyperglycemic Drugs on the Development of Chronic Kidney Disease in a Retrospective Cohort of Patients With Incident Diabetes: A Research Letter
Source: Can J Kidney Health Dis. 2025 Aug 22;12:20543581251365364. doi: 10.1177/20543581251365364 (PMC12374082; doi:10.1177/20543581251365364)
Supplement: sj-docx-1-cjk-10.1177_20543581251365364 – Supplemental material for The “Real-World” Effect of Anti-hyperglycemic Drugs on the Development of Chronic Kidney Disease in a Retrospective Cohort of Patients With Incident Diabetes: A Research Letter [file sj-docx-1-cjk-10.1177_20543581251365364.docx]

**Supplementary Methods:**

The institutional ethics review board at the University of Alberta (Pro00058561) approved this study and waived the requirements for participants to provide consent.

**Data source for the cohort:**

This retrospective cohort was created through data linkages between the ATP cohort^9^, which is a prospective cohort created with Alberta residents aged 35 to 69 and no history of cancer that collects comprehensive data on sociodemographic factors, personal lifestyle, environmental and socio-psychological factors, DNA and other biomarkers, and Alberta health (AH; the provincial health ministry) including data on registration, vital statistics, provider claims, hospitalizations, ambulatory care utilization, pharmaceutical information network (outpatient medications); and from the clinical laboratories in Alberta. All Alberta residents are eligible for insurance coverage by AH and >99% participate in coverage. We collected data from October 1^st^, 2000, to March 31^st^, 2021. We included adult patients with incident diabetes in the ATP cohort randomly recruited from Alberta’s adult general population in 2000 to 2008. Prescription of anti-diabetic drugs were identified using outpatient medication prescriptions.

**Exposures and Outcomes:**

Diabetes was defined using the National Diabetic Surveillance System (NDSS) and ATP cohort self-report. Incident cases were defined using an index date later than 6 months after recruitment after the ATP enrollment. The initial dispense date of anti-diabetic drugs within 6 months of diabetes diagnosis; excluded course end date in 6 months before diabetes diagnosis (diabetes unrelated use). SGLT-2i drug dosing described was based on the most frequently prescribed SGLT-2i (dapagliflozin). Common doses of dapagliflozin (either 5 or 10mg) was used as a rough estimate of the dose response effect for SGLT-2 inhibitors. Seventy-three of the 360 patients on SGLT-2 inhibitors were on dapagliflozin. A start date in 6 months before diagnosis were also excluded to reduce protopathic bias.

Chronic kidney disease was defined using a case definition of mean eGFR <60 mL/min*1.73 m2 (2010-18) or mean albuminuria >30 mg/g over 12 months or 1 hospitalization or 3 claims within 1 year (ICD-9: 583, 584, 585, 586, 592, 593.9, ICD-10: N00-N23). This case definition was used as the ATP cohort is limited by lab data from 2010-2018. Hemodialysis was defined as the following hospital claims: CCPX (ICD-9) = 13.99A, 13.99B, 13.99C, 13.99D, 13.99O, 13.99OA. Peritoneal dialysis was defined using the following hospital claims: 13.99C, 13.99D, 13.99O, 13.99OA. Kidney failure was defined by two hemodialysis sessions within 1 year but 90 days apart in claims. Kidney transplant was defined using the following claim codes: CCI = 1PC85 in DAD OR CCPX (ICD-9) = 67.59A. Kidney related cause of death was defined using a combination of ICD-10 (N0-N3) in vital statistics data.

**Statistical Analysis:**

The data analysis was completed using Stata MP 18.0 ([www.stata.com](http://www.stata.com)) and we reported baseline descriptive statistics as counts and percentages, or medians and inter-quartile limits, as appropriate. Hazard ratios and its 95% confidence intervals were calculated after adjusting for time related variation in use of oral anti-diabetic drugs, age at diabetes diagnosis, sex (male/female), ethnicity (European ancestry vs. other), living in rural vs. urban areas, education attainment (secondary or less, some post-secondary, post-secondary), BMI categories (<24.9 kg/m2, 25.0-29.9 kg/m2, >30.0 kg/m2 ), ever smoker (yes/no), number drinks of alcohol per day, physically active (yes/no, based on accumulating at least 210 min of moderate- to vigorous-intensity recreational physical activities per week in the past 12 months), tertiles of Healthy Eating Index Canada score for diet quality assessment, the number of Elixhauser comorbidities (0, 1-2, 2+ comorbidities) at diagnosis, time-varying measures of use of insulin and drugs for cardiovascular diseases (lipid-lowering agents, diuretics, beta blocker, calcium channel blocker, agents on renin-angiotensin system and mineralocorticoid receptor antagonists) and hypertension. High-dimensional propensity scores (HDPS) for oral anti-diabetic drugs; HDPS was calculated based on data using 43 different dimensions. Additional analysis was conducted on the post-2014 cohort (n=1435) to account for differences that may have occurred after the introduction of SGLT-2 inhibitors in 2014 and assess the effect of protopathic bias.

**Supplementary table 1: Breakdown of prescriptions of anti-hyperglycemic medications and outcomes**

|  |  | n (%) |  |
| --- | --- | --- | --- |
| Total Population | Incident Diabetes Cases | 3,001 |  |
| Use of anti-hyperglycemic medications |  |  |  |
|  | Metformin | 1, 749 (58.8) |  |
|  | SGLT-2i | 360 (12.0) |  |
|  | DPP-4i | 313 (10.4) |  |
|  | GLP-1Ra | 188 (6.3) |  |
|  |  |  |  |
| Number of co-morbidities in users of anti-hyperglycemic medications |  | 0-1 | 2+ |
|  | Metformin | 1,290 (73.8) | 459 (26.2) |
|  | SGLT-2i | 285 (79.2) | 75 (20.8) |
|  | DPP-4i | 238 (76.0) | 75 (24.0) |
|  | GLP-1Ra | 144 (76.6) | 44 (23.4) |
|  |  |  |  |
| Outcomes after diagnosis of diabetes (unadjusted events)) |  |  |  |
|  | Composite outcome | 628 (20.9) |  |
|  | Incident Chronic kidney disease | 512 (16.9) |  |
|  | Others kidney related events* | 116 (3.9) |  |

Abbreviations: DPP-4i: dipeptidyl peptidase-4 inhibitors; GLP-1Ra: glucagon-like peptide-1 receptor agonists; SGLT-2i: sodium-glucose cotransporter-2 inhibitors

*other kidney related events include abnormal estimated glomerular filtration rate, albuminuria, dialysis (hemodialysis/peritoneal dialysis), kidney failure, kidney transplant, and kidney related death^10^
